# Traditional Games as Cultural Heritage: The Case of Canary Islands (Spain) From an Ethnomotor Perspective

**DOI:** 10.3389/fpsyg.2021.586238

**Published:** 2021-02-25

**Authors:** Rafael Luchoro-Parrilla, Pere Lavega-Burgués, Sabrine Damian-Silva, Queralt Prat, Unai Sáez de Ocáriz, Enric Ormo-Ribes, Miguel Pic

**Affiliations:** ^1^Motor Action Research Group (GIAM), INDEST, National Institute of Physical Education of Catalonia (INEFC), University of Lleida, Lleida, Spain; ^2^Motor Action Research Group (GIAM), National Institute of Physical Education of Catalonia (INEFC), University of Barcelona, Barcelona, Spain; ^3^Motor Action Research Group (GIAM), Institute of Sport, Tourism, and Service, South Ural State University, Chelyabinsk, Russia

**Keywords:** intangible cultural heritage, motor praxeology, ethnomotricity, sustainability, relational well*-*being, material culture

## Abstract

UNESCO in the 2030 agenda for sustainable development establishes respect for the environment and sustainability education as key elements for the challenges of society in the coming years. In the educational context, physical education can have a vital role in sustainability education, through Traditional Sporting Games (TSG). The aim of this research was to study from an ethnomotor perspective the different characteristics of two different groups of TSG (with and without objects) in the Canary Islands, Spain. The corpus of this investigation was made up of 513 TSG, identified by two analysis techniques and collected in a database. The categories corresponding to the variables of the internal logic of the game were the type of motor interaction, related to space, relationships with time (competition), and relationships with objects. The study also examined the variables of external logic or sociocultural conditions such as the protagonists, playing areas, and game moments. The data analysis was carried out using descriptive and inferential statistics: cross-tables, effect sizes, classification trees (CHAID), and the identification of frequency areas. Of the total number of playful activities identified (*n* = 664), most were physical activities (*n* = 513/664; 77.26%) (non-physical activities: *n* = 151/664; 22.74%). These activities were Quasi-games without rules (*n* = 87) and TSG (*n* = 426) as well as activities with Objects (*n* = 299) and without material (*n* = 214). This research confirms that the TSG in the Canary Islands is a mirror of traditional culture and, from a pedagogical approach, shows great potential for material and social sustainability.

## Introduction

Almost 20 years ago, UNESCO (2003) defined intangible cultural heritage as “*The ‘intangible cultural heritage’ means the practices, representations, expressions, knowledge, skills – as well as the instruments, objects, artifacts, and cultural spaces associated therewith – that communities, groups and, in some cases, individuals recognize as part of their cultural heritage.”* According to this definition, Traditional Sporting Games (TSG) constitute a cultural heritage, since they are manifestations that are expressed through body language, that is, motor actions (e.g., Caillois, 2001; Sutton-Smith, 2001; Huizinga, 2010). The 2nd article of the recently passed Canarian Law on Physical Activity and Sport (Ley, 2019) states “*the right to know (…) and promote sport as an integral element of our culture, recovery, maintenance and development of autochthonous, and traditional sports and motor games (…), as an expression of our insular reality*” (Law 1/2019, of January 30th, on Physical Activity and Sport in the Canary Islands, 2019). TSG are the main asset of physical education to enhance the local culture. These traditional activities constitute a cultural legacy in the respectful way of relating to others and also to the environment. Moreover, UNESCO’s 2030 agenda (UNESCO, 2017) establishes “respect for the environment and sustainability education as key elements for the challenges of society.”

According to Parlebas(2001, p.286), TSG correspond to “*sporting games, frequently rooted in a long cultural tradition, which have not been regulated by official authorities.*” The rules of these games bear the distinctive characteristics of the local culture and show the great diversity that characterizes the immaterial recreational heritage. When an activity does not have rules, it means that the way of playing is very open, allowing the practice conditions to be continuously modified, the reason why these activities are called “quasi-games” (Parlebas, 2001). Each and every TSG has an internal logic that leads the agents to solve original problems associated with the relationships to the other participants, to space, to time, and to objects (Parlebas, 2001). However, when relating the internal logic of game to the culture it belongs to, it is necessary to consider aspects external to the rules of the game, but constitutive to the game situation such as the characteristics of the players (i.e., age and gender), the playground (i.e., indoors/outdoors, degree of arrangement), the temporal circumstances (i.e., season of the year), and the objects (i.e., origin and methods of production). This connection between internal logic and culture is captured in the notion of ethnomotricity, understood as “*field and nature of motor practices, considered from the point of view of their relationship with culture, and the social environment in which they have been developed*” (Parlebas, 2001, p. 227).

The gradual loss of different games of children and adults that were played on the islands in the mid-20th century onward makes us consider the importance of knowing, rescuing, and maintaining the different TSGs that were developed in each one of the islands (Navarro, 1994; Castro, 2001). Various studies in the Canary Islands (Navarro, 1994; Castro, 2001), and in other regions too (Lavega, 1995; Etxebeste, 2001; Warnier, 2001; Lavega et al., 2006; Hastie and André, 2012; Rauber et al., 2014; Parlebas, 2016a; Proyer et al., 2018; Lavega-Burgués and Navarro-Adelantado, 2019), have shown that TSG are cultural expressions that contain representative values of those communities. “*Entering a game is entering a society. A game is a kind of emblem of culture; therefore, deep knowledge of playful practices is an important element of the knowledge of a society*” (Parlebas, 2005, p. 13). Therefore, we can be certain that Traditional Sporting Games of the Canary Islands (TSGC) are a mirror of the local culture, traditions, and society of our archipelago (Sánchez and Suárez, 1996; Concepción, 2006; Hernández et al., 2015). These studies have provided evidence that through TSG, core social values can be promoted, such as learning to live together, social inclusion, gender equality, socio-emotional well-being, and sustainable actions related to the other people as well as the physical environment. In order to properly understand the Canary Islands’ playful activities, it is necessary to know that the Islands come from a volcanic origin, with characteristics that are unique in terms of climate, landscape, and vegetation compared to other regions of Spain. Moreover, they form an archipelago with a historically agricultural economy. The Canary Islands were a place of passage for migrants traveling to the Americas or for staying on the Islands. The migrants used to bring with them representative elements of their playful traditions. In this study, we are going to analyze children’s ludomotor activities with cultural local traits and with other similar features to other regions of the Spanish territory (Amades, 1984; Lavega et al., 2009; Cortizas, 2013; López and García, 2014).

The presence of objects in any TSG reveals a direct link with local material culture. Each and every player of these TSG is “shaped, subjectified by its embodied material culture” (Warnier, 2011). These elements often come from the local environment where the players find them, to prepare them in a unique way to be used in the game. “*The reuse of objects from the domestic environment and the ecological use of materials from nature confirm the condition of playful heritage of these manifestations*” (Lavega, 2017, p. 6). This is a clear example of a respectful and sustainable learning (Etxebeste et al., 2015).

The relatively non-existent research or specific publication on the comparison of games with and without objects has led us to delve into the distinctive features of these two great families of TSG. This study will facilitate the understanding of the TSG as a mirror of the society that has starred them (Navarro, 2002).

Taking into account this theoretical background, the objectives of this investigation were:

(a) To reveal from an ethnomotor perspective the distinctive features of TSGs with and without objects identified in the Canary Islands, Spain.

(b) To identify the predictive strength of the variables corresponding to the rules (internal logic) and their sociocultural context (external logic) to characterize the ethnomotor features of the TSG with and without objects in the Canary Islands (Spain).

## Materials and Methods

### Corpus

The original corpus of this investigation was made up of 664 games. Some of these TSG (151) were excluded from the study due to an unfulfillment of the motor skill or motor situation criterion (Parlebas, 2003, p. 67). Finally, 513 TSGs completed the total number of games susceptible to analysis.

### Ethnomotor Variables

Various categories corresponding to the variables of the internal logic of the game were considered: (a) *type of motor interaction:* psychomotor (Psyco), cooperation (Coop), opposition (Oppo), and cooperation–opposition (CoopOppo); (b) *relation to space:* stable (Stab) or unstable (Unstab); (c) *relation with time:* with competition (Acco) and without competition (Nonacco); and (d) *relation with objects:* with objects (Wobj) and without objects (Nobj); as well as variables of external logic or sociocultural conditions: (a) *protagonists age*: infant (Infa), juvenile (Yout), adult (Adul), and all ages (Alla), and protagonist sex: masculine (Mas), feminine (Fem), and mixed (Mix); (b) *play areas (type of facilities)*: internal (Int), external (Ext), and internal–external (IntExt); playing areas (preparation): prepared (Prep), little prepared (Litprep), and not prepared (Nonprep); and (c) *game moments*: with calendar (Cale) and without calendar (Noncale)^1^.

### Instruments and Procedure

The TSGs were identified using two analysis techniques: (a) content analysis of 27 written consultation sources, published in the Canary Islands, from 1940 onward (Diego, 1943; Hernández, 2005); (b) TSG compilation using oral sources and materials, collected by various researchers for more than 20 years and complemented by semi-structured interviews with six players and artisans of game objects (Lagardera et al., 2018). A guideline was designed with short, open-ended questions concerning aspects of the rules of the games and sociocultural conditions of practice. Data collection was carried out through video and audio recordings and field notes that provided a complement to this research.

There were four observers specialized in traditional games with more than 10 years of experience in the “*ad hoc*” construction of the registration system, and all games were classified by consensus.

### Data Analysis

Through the use of the statistical software SPSS v.25, it was possible to determine and complement the previous ethnomotor analysis. Parlebas (2016a, p.46) points out “Each game is a universe that makes cultural sense and must be interpreted on the basis of the observed motor sequences, on the configurations and the networks discovered.” According to this approach, we also used frequency areas technique to identify the ethnomotor chains of the traditional Canarian games. These descriptive and multimodal graphs facilitate the comparison of games with and without objects in the set of ethnomotor variables studied (≥3 numbers of games with or without objects).

Descriptive and inferential statistical techniques were used: cross-tables, effect sizes, classification trees, and identification of frequency areas.

Statistical margins were used for all analysis (*p* < 0.05). Cross-tables were generated with Pearson’s chi-squared values, as well as adjusted residuals (ARs) between the margins (ARs) >1.96 or <−1.96. Subsequently, the effect size was calculated using Cramer’s *V* test (0.10 = small effect, 0.30 = medium effect, and 0.50 = large effect) (Field, 2013).

An exhaustive chi-squared automatic interaction detector (CHAID) classification tree (Ye et al., 2016; Lorenzo et al., 2017) was used to determine the dependent variable (with and without objects) regarding the rest of the predictive variables. The following requirements were used to build the model: (i) Pearson’s chi-squared test was applied; (ii) a maximum of five depth levels; (iii) margin of cases between 50 and 100 between the nodes; and (iv) cross-validation, consisting of randomizing and dividing the data up to 10 times, with 90% of the total cases used for learning the model, while 10% of the total was reserved for the final test (Thornton et al., 2016).

## Results

Most of the playful practices analyzed (*n* = 664) were motor in nature (513 motor = 77.26%; 151 non-motor; 22.74%).

### Rules in Playful Practices With and Without Objects

Most of the recreational practices (*n* = 513) corresponded to TSG, that is, activities created by rules that established rights and prohibitions to be respected by the players. Significant differences (*p* < 0.001; *ES* = 0.340) (Table 1) were found depending on the variable of the rules variable. The quasi-games (Alga) (Table 2) were carried out mostly with objects (*n* = 83; *Ar* = 7.7; 16.2%) with respect to the activities without object (*n* = 4; *Ar* = −7.7; 0.8%). Regarding games (Traga), there was a slight predominance of games with object (*n* = 216; *Ar* = −7.7; 42.1%) in relation to regulated activities without object (*n* = 210; *Ar* = 7.7; 40.9%).

**TABLE 1:** Results of cross-tables taking into account internal and external logic variables with and without objects.

Logic	Variables	Categories	With objects		Without objects	
			*n;* %	*Ar*	*n;* %	*Ar*
Internal logic variables	Rule (*p* < 0.001; *ES* = 0.340)	Quasi-Game	*n* = 83 16.2%	7.7	*n* = 4 0.8%	−7.7
		Sporting Game	*n* = 216 42.1%	−7.7	*n* = 210 40.9%	7.7
	Motor interactions (*p* < 0.001; *ES* = 0.571)	Psychomotor	*n* = 136 26.5%	11.1	*n* = 3 0.6%	−11.1
		Opposition	*n* = 81 15.8%	1.2	*n* = 48 9.4%	−1.2
		Cooperation–Opposition	*n* = 26 5.1%	1	*n* = 18 3.5%	−1
		Cooperation	*n* = 56 10.9%	−11.2	*n* = 145 28.3%	11.2
	The game space (*p* < 0.001; *ES* = 0.157)	Stable	*n* = 282 55%	−3.5	*n* = 214 41.7%	3.5
		Unstable	*n* = 17 3.3%	3.5	*n* = 0 0%	−3.5
	Scoring system (result) (*p* < 0.001; *ES* = 0.307)	With scoring	*n* = 77 15%	7	*n* = 6 1.2%	−7
		No Scoring	*n* = 222 43.3%	−7	*n* = 208 40.5	7
External logic variables or sociocultural conditions	Age of the players (*p* < 0.001; *ES* = 0.214)	Children	*n* = 247 48.1%	−4.7	*n* = 206 40.2%	4.7
		Youth	*n* = 17 3.3%	2.2	*n* = 4 0.8%	−2.2
		Adult	*n* = 26 5.1%	3.2	*n* = 4 0.8%	−3.2
		Any Age	*n* = 9 1.8%	2.6	*n* = 0 0%	−2.6
	Gender of the protagonists (*p* < 0.001; *ES* = 0.317)	Female	*n* = 66 12.9%	−8.1	*n* = 122 23.8%	8.1
		Male	*n* = 78 15.2%	5.1	*n* = 18 3.5%	−5.1
		Mixed	*n* = 155 30.2%	3.9	*n* = 74 14.4%	−3.9
	The location of practice areas (*p* < 0.001; *ES* = 0.263)	External	*n* = 167 32.6%	−5.7	*n* = 171 33.3%	5.7
		Internal	*n* = 10 1.9%	−2	*n* = 8 1.6%	2
		Internal–External	*n* = 122 23.8%	5.9	*n* = 35 6.8%	−5.9
	Preparation of zones in games (*p* < 0.001; *ES* = 0.299)	Not prepared	*n* = 225 43.9%	−6.8	*n* = 208 40.4%	6.8
		Little prepared	*n* = 20 3.9%	3.5	*n* = 1 0.2%	−3.5
		Prepared	*n* = 54 10.5%	5.5	*n* = 5 1%	−5.5
	Calendar in the games (*p* < 0.005; *ES* = 0.123)	No Calendar	*n* = 285 55.6%	−2.8	*n* = 213 41.5%	2.8
		Calendar	*n* = 14 2.7%	2.8	*n* = 1 0.2%	−2.8

**TABLE 2:** Different play chains of the frequency area with or without objects.

		ILobject		Total
		Nobj	Wobj	
Alga,Psycho,Stab,Nonacco,Infa,Mas,Ext,Nonprep,Noncale	A	0	3	3
Alga,Psycho,Stab,Nonacco,Infa,Mas,IntExt,Nonprep,Noncale	B	0	15	15
Alga,Psycho,Stab,Nonacco,Infa,Mix,Ext,Nonprep,Noncale	C	0	7	7
Alga,Psycho,Stab,Nonacco,Infa,Mix,IntExt,Nonprep,Noncale	D	0	36	36
Alga,Psycho,Unstab,Nonacco,Infa,Mix,Ext,Nonprep,Noncale	E	0	5	5
Traga,Coop,Stab,Nonacco,Adul,Mix,Int,Nonprep,Noncale	F	2	1	3
Traga,Coop,Stab,Nonacco,Infa,Fem,Ext,Nonprep,Noncale	G	97	45	142
Traga,Coop,Stab,Nonacco,Infa,Fem,IntExt,Nonprep,Noncale	H	23	4	27
Traga,Coop,Stab,Nonacco,Infa,Mas,Ext,Nonprep,Noncale	I	9	0	9
Traga,Coop,Stab,Nonacco,Infa,Mix,Ext,Nonprep,Noncale	J	8	0	8
Traga,Coop,Stab,Nonacco,Yout,Mix,Int,Nonprep,Noncale	K	3	0	3
Traga,CoopOppo,Stab,Acco,Infa,Mix,Ext,Nonprep,Noncale	L	0	4	4
Traga,CoopOppo,Stab,Acco,Infa,Mix,Ext,Prep,Noncale	M	2	4	6
Traga,CoopOppo,Stab,Acco,Yout,Mix,Ext,Litprep,Noncale	N	0	3	3
Traga,CoopOppo,Stab,Nonacco,Infa,Mas,Ext,Nonprep,Noncale	O	3	3	6
Traga,CoopOppo,Stab,Nonacco,Infa,Mix,Ext,Nonprep,Noncale	P	10	1	11
Traga,Oppo,Stab,Acco,Infa,Mas,Ext,Litprep,Noncale	Q	0	3	3
Traga,Oppo,Stab,Acco,Infa,Mas,IntExt,Prep,Noncale	R	0	10	10
Traga,Oppo,Stab,Acco,Infa,Mix,Ext,Nonprep,Noncale	S	1	2	3
Traga,Oppo,Stab,Acco,Infa,Mix,IntExt,Nonprep,Noncale	T	1	3	4
Traga,Oppo,Stab,Acco,Infa,Mix,IntExt,Prep,Noncale	U	0	4	4
Traga,Oppo,Stab,Acco,Yout,Mix,Ext,Litprep,Noncale	W	0	3	3
Traga,Oppo,Stab,Nonacco,Infa,Mas,Ext,Nonprep,Noncale	X	3	2	5
Traga,Oppo,Stab,Nonacco,Infa,Mas,IntExt,Nonprep,Noncale	Y	2	2	4
Traga,Oppo,Stab,Nonacco,Infa,Mix,Ext,Nonprep,Noncale	Z	30	10	40
Traga,Oppo,Stab,Nonacco,Infa,Mix,Ext,Prep,Noncale	AA	1	3	4
Traga,Oppo,Stab,Nonacco,Infa,Mix,IntExt,Nonprep,Noncale	BB	6	7	13
Traga,Psycho,Stab,Acco,Infa,Mas,Ext,Prep,Noncale	CC	0	3	3
Traga,Psycho,Stab,Nonacco,Adul,Mas,Ext,Nonprep,Noncale	DD	0	3	3
Traga,Psycho,Stab,Nonacco,Infa,Fem,IntExt,Nonprep,Noncale	EE	0	13	13
Traga,Psycho,Stab,Nonacco,Infa,Mix,Ext,Prep,Noncale	FF	0	3	3
Traga,Psycho,Stab,Nonacco,Infa,Mix,IntExt,Nonprep,Noncale	GG	0	13	13
		214	299	513

### Motor Communication in Games With and Without Objects

Significant differences were found (*p* < 0.001; *ES* = 0.571), when comparing both types of activities depending on the type of motor interaction. Most psychomotor practices were carried out with objects (*n* = 136; *Ar* = 11.1; 26.5%) [psychomotor games (Psycho) without objects: *n* = 3; *Ar* = −11.1; 0.6%]. When the games were performed with the presence of opponents, the presence of objects predominated: Opposition games (Oppo) with objects (*n* = 81; *Ar* = 1.2; 15.8%) vs. TSG without objects (*n* = 48; *Ar* = −1.2, 9.4%); cooperation–opposition games (CoopOppo) with objects (*n* = 26; *Ar* = 1; 5.1%) and without objects (*n* = 18; *Ar* = −1; 3.5%). On the other hand, this regularity occurred in the opposite direction in the cooperation games (Coop) in which the presence of activities without objects predominated (*n* = 145; *Ar* = 11.2; 28.3%) in relation to the games with objects (*n* = 56; *Ar* = −11.2; 10.9%).

### The Stable or Unstable Spaces Used by Games With and Without Objects

Significant differences were found (*p* < 0.001; *ES* = 0.157) when comparing both types of activities depending on the stable or uncertainty related to the space where games were played. When the activities were carried out in a stable space, both types of games had a more balanced distribution, with predominance of games with objects in stable spaces without objects (*n* = 214; *Ar* = 3.5; 41.7%) and stable spaces with objects (*n* = 282; *Ar* = −3.5; 55%). If the games were performed in an unstable space, bearing uncertainty, they were only performed with the presence of objects: unstable space without objects (*n* = 0; *Ar* = −3.5; 0%) and unstable space with objects (*n* = 17; *Ar* = 3.5; 3.3%).

### Score (Result) in Games With and Without Objects

Significant differences were found (*p* < 0.001; *ES* = 0.307) when comparing both types of activities based on the result or accounting on the scoring system. Most games with a scoring system were performed with objects (*n* = 77; *Ar* = 7; 15%) compared to games without objects (*n* = 6; *Ar* = −7; 1.2%). When there was no competition among the participants, there was a slight superiority of games with objects (*n* = 222; *Ar* = −7; 43.3%) in relation to games without objects (*n* = 208; *Ar* = 7; 40.5%).

In relation to the distinctive features of the variables of external logic or sociocultural conditions, the following results were found.

### Age of the Players in Games With or Without Objects

Significant differences were found (*p* < 0.001; *ES* = 0.214) when comparing both types of activities based on the age of the participants. In childhood, there was a slight superiority of games with objects (*n* = 247; *Ar* = −4.7; 48.1%) compared to games without material (*n* = 206; *Ar* = 4.7; 40.2%). In adolescence (Yout), games with objects predominated (*n* = 17; *Ar* = 2.2; 3.3%) over games without objects (*n* = 4; *Ar* = −2.2; 0.8%). Adult games with objects (*n* = 26; *Ar* = 3.2; 5.1%) also predominated in relation to games without material (*n* = 4; *Ar* = −3.2; 0.8%). Games that could be performed at any age (Alla) were only played with objects (*n* = 9; *Ar* = 2.6; 1.8%) (games without objects: *n* = 0; *Ar* = −2.6; 0%).

### Gender of the Protagonists in Games With and Without Objects

Significant differences were found (*p* < 0.001; *ES* = 0.317) when comparing both types of activities according to gender. The female gender (Fem) used mostly games without objects (*n* = 122; *Ar* = 8.1; 23.8%), compared to games with objects (*n* = 66; *Ar* = −8.1; 12.9%). This regularity was reversed in the games of the masculine gender (Mas): Games with objects (*n* = 78; *Ar* = 5.1; 15.2%); games without objects (*n* = 18; *Ar* = −5.1; 3.5%). Mixed games (Mix) also reflected a greater presence of activities with objects (*n* = 155; *Ar* = 3.9; 30.2%) than without material (*n* = 74; *Ar* = −3.9; 14.4%).

### The Location of Practice Areas in Games With and Without Objects

Significant differences were found (*p* < 0.001; *ES* = 0.263) when comparing both types of activities depending on the playing area. When games were played in outdoors (Ext) there was a slight superiority of games without objects (*n* = 171; *Ar* = 5.7; 33.3%), compared to games with objects (*n* = 167; *Ar* = −5.7, 32.6%). In games played indoors, this slight difference changed direction in favor of games with objects (*n* = 10; *Ar* = −2; 1.9%) compared to activities without material (*n* = 8; *Ar* = 2; 1.6%). Finally, games performed both indoors and outdoors (IntExt) were mostly games with objects (*n* = 122; *Ar* = 5.9; 23.8%) compared to activities without objects (*n* = 35; *Ar* = −5.9; 6.8%).

### Preparation of Zones in Games With and Without Objects

Significant differences were found (*p* < 0.001; *ES* = 0.299) depending on the type of conditioning of the playgrounds. Most of the games with and without objects were performed in unprepared areas (Nonprep) (*n* = 433; 84.4%). Under these conditions, there was a slight superiority of games with objects (*n* = 225; *Ar* = −6.8; 43.9%), compared to games without material (*n* = 208; *Ar* = 6.8; 40.4%). When the areas were little prepared (Litprep), games with objects predominated (*n* = 20; *Ar* = 3.5; 3.9%) compared to those that did not use materials (*n* = 1; *Ar* = −3.5; 0.2%). Finally, if the games were made in a specific facility to play (Prep), the games were mostly with objects (*n* = 54; *Ar* = 5.5; 10.5%) in relation to games without objects (*n* = 5; *Ar* = −5.5; 1%).

### The Calendar in Games With and Without Objects

Significant differences were found (*p* < 0.005; *ES* = 0.123) depending on the type of conditioning of the playing areas. The vast majority of games were played at any time of the year, without following a calendar (*n* = 498; 97.1%). This type of activities was dominated by games with objects (*n* = 285; *Ar* = −2.8; 55.6%) in relation to games without material (*n* = 213; *Ar* = 2.8; 41.5%). When games had a calendar, games with objects also predominated (*n* = 14; *Ar* = 2.8; 2.7%) compared to games without material (*n* = 1; *Ar* = −2.8; 0.2%).

### Predictive Capacity of the Ethnomotor Variables (of the Internal Logic and the External Logic) of the TSGC With and Without Material

**FIGURE 1:**
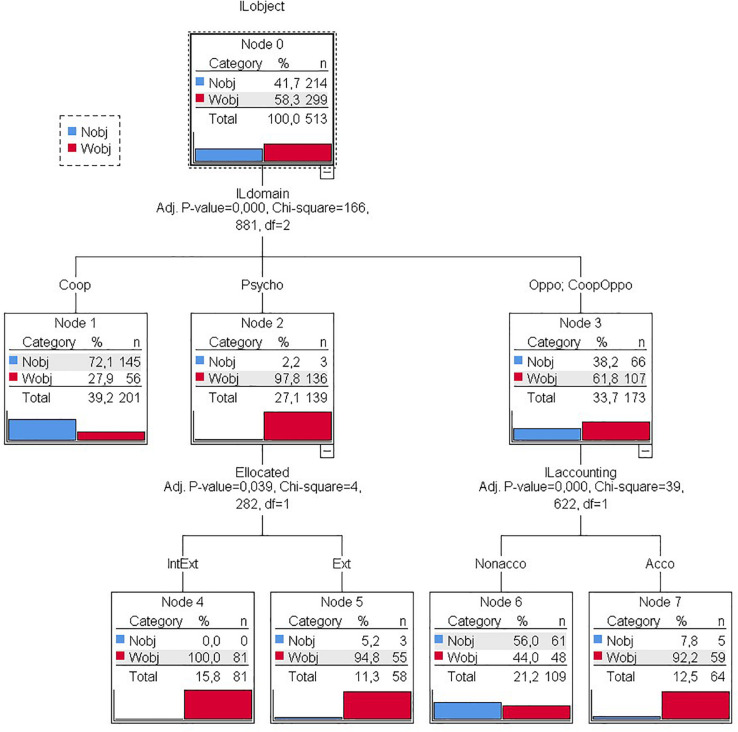
Decision tree of various variables with or without game objects.

The exhaustive CHAID model or decision tree showed a proportion of 78% correctly classified cases with a standard error (misclassification risk) of 0.018 (Figure 1).

The variable with the highest predictive strength was the type of motor interaction. Significant differences (*p* < 0.001; χ^2^ = 166.881; *df* = 2) were found between psychomotor games (node 2), cooperation games (node 1), and games with adversaries (node 3), opposition or cooperation–opposition. As observed in the previous type of statistical analysis, games with objects predominated in psychomotor games and with the presence of an opponent, unlike cooperative games in which games without objects predominated.

The location of the playing area (nodes 4 and 5) was the only predictive variable for psychomotor games. Significant differences (*p* = 0.039; χ^2^ = 4.282; *df* = 1) were found between games that were played in outdoors (node 5) and those that were played in indoors or outdoors (node 4). In both cases, there was a clear predominance of games with objects.

For games with an opponent (opposition and cooperation–opposition), score-keeping was the only predictive variable shown by the classification tree. There were significant differences (*p* < 0.001; χ^2^ = 39.622; *df* = 1) in games with and without competition (a score system). The games with a scoring system were mostly with objects, while in games without competition, the distribution was similar, with a slight predominance of games without objects.

### A Multidimensional View of Games With and Without Objects

**FIGURE 2:**
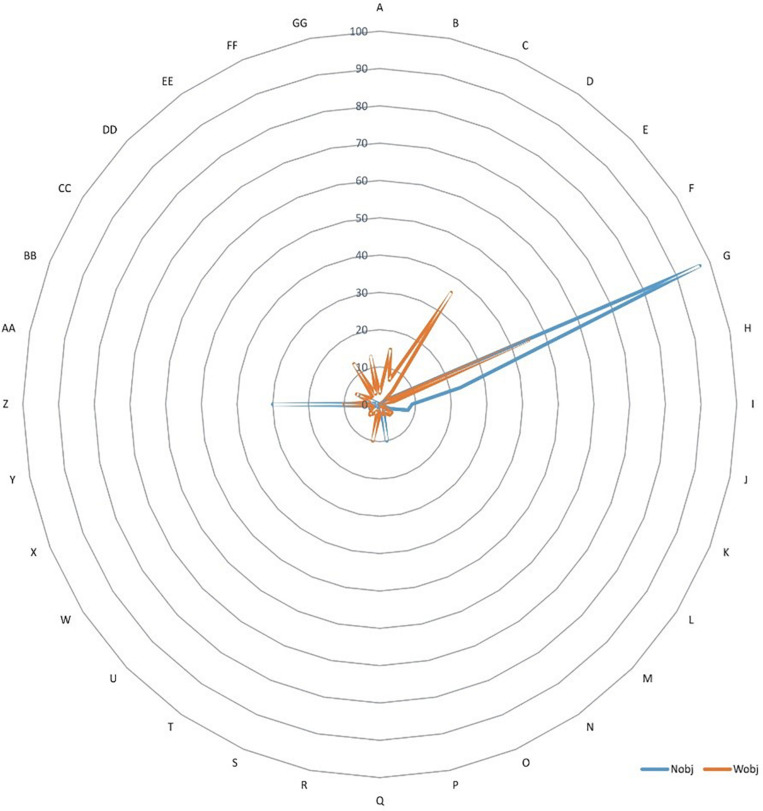
Frequency area of various variables with or without game objects.

The largest frequency areas (Figure 2) were G (Traga, Coop, Stab, Nonacco, Infa, Fem, Ext, Nonprep, Noncale), with a result of games without blue objects (*n* = 97) and games with red colored objects (*n* = 45). The H frequencies (Traga, Coop, Stab, Nonacco, Infa, Fem, IntExt, Nonprep, Noncale) stand out, with (*n* = 23) blue color and (*n* = 4) red color, in addition to D with (*n* = 35) exclusively red and the Z (Traga, Oppo, Stab, Nonacco, Infa, Mix, Ext, Nonprep, Noncale) with (*n* = 30) blue color and (*n* = 10) red color that demonstrate the predominance of this type of game in this frequency area.

## Discussion

This study attempted to reveal from an ethnomotor perspective the distinctive features of TSGs with and without objects identified in the Canary Islands, Spain. It was also proposed to identify the predictive strength of the variables corresponding to the rules (internal logic) and their sociocultural context (external logic) to characterize the ethnomotor features of the TSG with and without objects in the Canary Islands (Spain).

This study should serve as the basis for building new educational projects in the Canary Islands that address the challenges necessary for the 21st century, such as democratic coexistence or sustainability. For this, it is necessary to delve into the ethnomotor footprint, an objective that characterizes the legacy of the traditional Canarian leisure culture.

The ethnomotor approach (Parlebas, 2001; Lavega-Burgués and Navarro-Adelantado, 2019) has revealed the distinctive features of the internal and external logics of the Canary Islands, TSG in their condition of cultural heritage (Parlebas, 2016b, 2017; Navarro-Adelantado and Lavega, 2017; Lavega, 2018; UNESCO, 2018).

### Ethnomotor Diversity

Canarian culture offers a wide variety of playful activities that testify to the integrative nature of its games. The analysis carried out identified several ethnomotor trait-chain activities: (a) with rules (specific conditions of the game are established); (b) with a diversity of motor relations (especially cooperative games); (c) predominance of the use of material (objects from the immediate environment); (d) with or without a score system (which allowed comparing the results of the participants or playing games without counting the successes of the players); (e) children’s games (they are games mainly intended for the first ages); (f) masculine, feminine, or mixed (both genders can enjoy the recreational culture); (g) carried out in a stable, interior–exterior space, often not prepared (a circumstance that favors adaptation to any circumstance); and (h) without calendar (made at any time).

This ethnomotor vision reveals some peculiarities of the Canarian games in relation to other features identified in other cultural contexts. In the Canary Islands, a high percentage granted to cooperation is observed (39%) compared to studies in which cooperation was residual, around a third of rivalry (Parlebas, 2001; Pic, 2018), and even lower values of 6.4% (Alonso et al., 2010) and 8.33% (Lavega and Navarro, 2015). This distribution is also different compared to events such as the Vitoria-Gasteiz International Games Festival (Alonso et al., 2010) or the Olympic Games (Pic, 2018; Parlebas, 2020).

### Games With and Without Objects Are Two Representative Families of the Canary Islands

Among the first findings, we highlight the existence of two large families of TSG, with the presence or absence of objects. Two internal logic variables [type of motor relationship (domain) and the score system] and one of the external logic game zone are the main predictors of games with and without material.

The games with objects are mainly psychomotor that can be performed in indoor or outdoor areas indifferently. These objects are usually obtained from the predominant vegetation or natural elements on the islands to make animal or people figures: (a) the verol (*Kleinia neriifolia* Haw), to make cows and human figures; (b) the gamona (*Asphodelus albus*), to create goats and mills collected in the South of Tenerife; (c) the Canary Island palm (*Phoenix canariensis*), a standard of the native flora and used to make bows, arrows, and mills; and (d) the shells and fossils, to make camels or barrels. Other playing objects are also made, such as stone throwers and spinning tops with native plants such as Drago, Sabina, Barbuzano, Acebuche, or Mocan.

The objects are also present in games of adversaries (opposition or cooperation–opposition), in this case associated with a scoring system. These are mainly games in which two people face each other (opposition duels) or two teams (cooperation–opposition duels). Some examples of objects used in these sociomotor games are banana leaves to build balls; the Canary Island palm tree (*P. canariensis*) was used to make sticks in team games such as *pina* (close to hockey) or *billarda* (kit-cat).

In these contexts, the objects are mediators of the ludic adventures since they give the opportunity to all the players to show their in the use of the game materials. Even when there are no rules, the objects favor the curiosity and motor creativity of the participants (Hüttermann et al., 2019), by constructing them in a personalized way and using them in a wide variety of playful situations (Casey and Hastie, 2011). The use of objects ensures that each player shapes unique experiences of embodied material culture (Warnier, 2011).

The games without objects are mainly cooperative, practiced mainly by girls. These activities are represented by circle games, in stable spaces and without a final score that favors interpersonal relations, although they can also be in games with adversaries (tagging games) performed in mixed groups (girls and boys) when there is no scoring system. In this case, the strength of the interpersonal relationship takes precedence over expertise in the use of game objects.

### Carpe Diem

An ethnomotor feature of the Canary games. The Canary Islands offers a great diversity of games that do not require a calendar. Surely having a moderate climate throughout the year favors playing at any time. Furthermore, on many occasions, the games do not have a final outcome (a scoring system), especially when they are cooperative; attention is directed to the development of the game itself (Etxebeste, 2001). From this TSG approach, give strength to the present time, to the enjoyment of each moment, giving meaning to the expression “carpe diem” through the TSG (Fernández et al., 2020).

The findings identified in this study provide educators with ethnomotor keys of interest to favor contextualized educational programs. The education of interpersonal relationships (Pic et al., 2018), sustainability, as well as learning to live with intensity every moment is a fundamental knowledge offered by the legacy of the TSG in the Canary Islands, as an intangible cultural heritage.

## Limitations

This study had a series of limitations and future prospects for other research groups. The increase in the number of categories was always a tempting possibility to gain precision. However, our main limitations were related to the search for evidences that confirmed the presence of recreational archeology in the Canarian culture. On the other hand, addressing the transfer of the findings found to a pedagogical level would have completed a cycle of research and action. Finally, other research groups could start from the motor situation. The study of girls and boys when playing would be to propose an enriching look to know in depth not only the cultural baggage hidden in TSGC but also the ethnomotor consequences resulting from playing and its scarce neutrality in practice.

## Future Perspective

The different possibilities that this study shows us, in the use of game objects in physical education classes, could be included in the educational curriculum of the Canary Islands, as a specific part of the promotion of the TSGC, being in tune with the context and not simply leaving it to the will and preferences of the teachers of the educational centers.

## Conclusion

This study provides an understanding of the ethnomotor traits of TSG in the Canary Islands as a way to shape original embodied material culture. The Canarian playful heritage has created two large families of ludomotor activities represented by two chains of ethnomotor traits: (a) With Objects: Quasi-Games, Psychomotor, Stable Space, without a final score, played by male–female children, in indoors–outdoors not specific to playgrounds, and without a calendar; (b) Without Objects: TSG, Cooperation, Stable Space, without a final score, played by female children, outdoors not specific to playgrounds, and without a calendar.

## Data Availability Statement

The original contributions presented in the study are included in the article/supplementary material, further inquiries can be directed to the corresponding author.

## Author Contributtons

RL-P, PL-B, and MP: substantial contribution to study conception and design. RL-P, PL-B, SD-S, QP, US, EO-R, and MP: preparation of the document for approval by the ethics committee, database revision, and writing of the manuscript. RL-P, PL-B, US, and MP: preparation and participation in the empirical work. RL-P, PL-B, EO-R, QP, and MP: discussion of the data analysis strategies. All authors contributed to the article and approved the submitted version.

## Conflict of Interest

The authors declare that the research was conducted in the absence of any commercial or financial relationships that could be construed as a potential conflict of interest. The reviewer MA declared a shared affiliation with two of the authors US and QP to the Editor at time of review.

## Footnotes

1 The description of these variables is available at http://praxiologiamotriz.inefc.es/?p=1333
